# Fabrication of Needle-Like Silicon Nanowires by Using a Nanoparticles-Assisted Bosch Process for Both High Hydrophobicity and Anti-Reflection

**DOI:** 10.3390/mi12091009

**Published:** 2021-08-25

**Authors:** Zengxing Zhang, Guohua Liu, Kaiying Wang

**Affiliations:** 1 Department of microsystems, University of South-Eastern Norway, 3184 Horten, Norway; zengxingzhang88@163.com; 2 Beijing Key Laboratory of Multiphase Flow and Heat Transfer for Low Grade Energy Utilization, North China Electric Power University, Beijing 102206, China; liuguohua126@126.com

**Keywords:** silicon nanowires, nanoparticles, Bosch process, anti-reflection, hydrophobicity

## Abstract

In this work, a modified Bosch etching process is developed to create silicon nanowires. Au nanoparticles (NPs) formed by magnetron sputtering film deposition and thermal annealing were employed as the hard mask to achieve controllable density and high aspect ratios. Such silicon nanowire exhibits the excellent anti-reflection ability of a reflectance value of below 2% within a broad light wave range between 220 and 1100 nm. In addition, Au NPs-induced surface plasmons significantly enhance the near-unity anti-reflection characteristics, achieving a reflectance below 3% within the wavelength range of 220 to 2600 nm. Furthermore, the nanowire array exhibits super-hydrophobic behavior with a contact angle over ~165.6° without enforcing any hydrophobic chemical treatment. Such behavior yields in water droplets bouncing off the surface many times. These properties render this silicon nanowire attractive for applications such as photothermal, photocatalysis, supercapacitor, and microfluidics.

## 1. Introduction

Silicon nanowires have been widely used over the past years in many emerging fields, including photodetection [1,2], photocatalysis [3,4], thermoelectric [5,6], and quantum information processing [7,8]. Nanowires are one-dimensional materials that present the shape of needles, rods, or pillars [9,10]. Their diameter lies in the range of nanometers to a few hundred nanometers [11], while their height can be as large as several micrometers [12]. Apart from the bottom-growth approaches, various patterning and dry etching processes have also been developed to realize nanowire fabrication with semiconductors [13,14,15]. Interestingly, most of the reported nanowire arrays are patterned by using electron-beam and holographic lithography techniques [16], which are attractive in generating a periodic pattern with a high degree of uniformity. However, the approaches mentioned above are time-consuming for large area nanowire fabrication and exhibit low throughput for mass production. As far as dry etching is concerned, it generally involves mask materials such as photoresist, silicon dioxide, and Teflon microspheres to fabricate structures with high aspect ratio profiles [16]. For instance, the employment of metal masks requires the enforcement of an additional lift-off treatment that decreases the pattern resolution and increases the process complexity. As a result, a specific eliminating step is needed that can cause irreducible contamination and damage. Recently, a self-assembled mask approach has emerged that is expected to address the issues mentioned above [17,18]. The method refers to forming a layer of gold NPs, which is formed by depositing a thin gold film. The subsequent application of a thermal annealing treatment to aggregate to be a particle-like hard mask without enforcing any other lithography procedures.

The frequently used etching treatments include laser ablation [19], metal-catalytic chemical etching [20,21], and reactive ion etching (RIE) [12,22]. Laser ablation is a random etching that can cause undercutting of the mask. The silicon wafer’s crystal orientation binds the catalyst-assisted etching. In contrast, deep reactive ion etching (DRIE) is the highly anisotropic etching method used to create holes and trenches with high aspect ratios and close-to-vertical sidewalls on various silicon substrates [17]. The Bosch process [23,24,25] is named after the invention of two employees of the German Robert Bosch company and its name originates from the company name. This process is also known as pulsed- or time-multiplexed-etching and includes the cycle between SF_6_ etching and C_4_F_8_ passivation, each phase lasting a few milliseconds. The passivation layer protects the hole/trench sidewalls from excessive bombardment. At the same time, the directional fluorine/sulfur ions bombard and remove the bottom passivation layer faster than that along the sidewalls. Such etch/protect steps alternate in sequence resulting in minimal isotropic etch taking place only at the bottom of the etched patterns, which makes the Bosch process become a tremendous potential method in nanostructure fabrication.

A novel one-step Bosch process is presented in this work to fabricate silicon nanowires with controllable surface density and a high aspect ratio. Our approach contains three main processing steps: NPs deposition as a mask material, modified Bosch etching, and removal of the mask. It is also attainable to change the nanowire’s structural profile by tuning the mask size and total etching duration. The modified Bosch process can transfer the NPs-based mask pattern into the silicon substrate and remove the particles in the etching process. In addition, the NPs-based mask is created by employing the magnetron sputtering for the deposition of a thin gold film (thickness from 4 to 10 nm) and subsequent thermal annealing at 500 °C for 1 h. The annealing temperature, holding time, and film thickness are crucial parameters to control the particle surface density and size. As a result, the enforcement of a higher annealing temperature, elevated holding times, and the employment of a thicker film led to a low surface density of the NPs and decreased filling density of the nanowires at last. In addition, our unique silicon nanowire arrays exhibit extremely superhydrophobic behavior with a contact angle over ~165.6° without any hydrophobic chemical treatment. Such behavior permits the water droplets to bounce off the surface many times, making it extremely difficult to conduct pendant-drop measurements. Besides, the fabricated silicon nanowire structure displays lower than 2% near-unity reflectance properties within an optical wave range of 220 to 1100 nm. It is interesting to notice that after incorporating the Au NPs and inducing the surface plasmons effect on their surface, a near-unity reflectance factor below 3% in the broadband range of 220 to 2600 nm is achieved.

## 2. Materials and Methods

### 2.1. Fabrication Process

**Figure 1:**
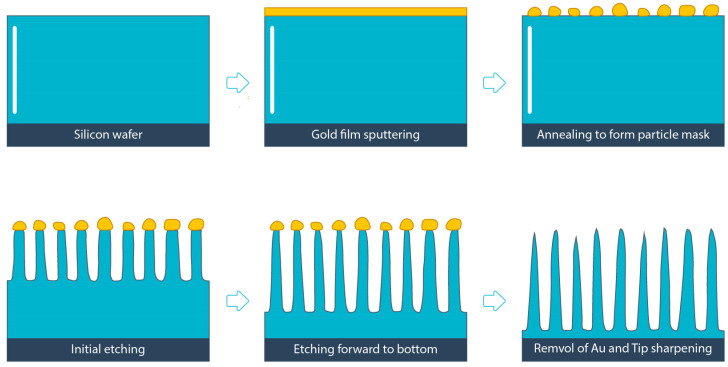
The schematic diagram of the nanowire fabrication process. The gold thin film is deposited on the P-type silicon wafer. Thermal annealing is employed to create gold particle mask. The followed etching treatment consists of initial Bosch etching, forward etching for more undercut, and the continue etching to remove Au nanoparticles.

The involved fabrication processes are schematically illustrated in Figure 1. Firstly, polished (100)-oriented p-type silicon wafers with a resistivity of 1–20 Ω·cm and thickness of 500 ± 10 µm were selected to fabricate the silicon nanowires. Next, all wafers were cleaned using a buffered oxide etch (BOE) solution at room temperature, followed by acetone, isopropanol, and deionized water (DI water) rinsing to remove the native oxide, residuals, and organic contamination. Next, gold thin films with various thicknesses of 4, 6, 8, and 10 nm were deposited on the wafer surface by employing the magnetron sputtering technique at room temperature (DC sputtering system, AJA International, Scituate, MA, USA). Next, the Au NPs-based masks were created by applying a rapid thermal annealing step at 500 °C for one hour. The Au NPs size presents a strong dependence on the film thickness. Subsequently, the silicon wafer was DRIE-etched with a modified Bosch process to create the nanowires. The etching process was conducted within an RIE cluster reactor (Plasma Pro100 Cluster, Oxford Instruments, Abingdon, UK).

### 2.2. Etching Conditions

The Bosch process is carried out through the fast alternative between two modes, namely the bombardment reaction by SF_6_ plasma and the protection by polymer C_4_F_8_ to obtain structures with nearly vertical sidewalls. Table 1 illustrates the modified etching recipe based on the Bosch process. The execution of such etching experiments requires the utilization of a helicon antenna-coupled plasma reactor that operates in the inductive mode. With the assistance of turbine vacuum pumps, we can rapidly and precisely adjust various experimental parameters such as gas flux, pressure, and plasma density. The employed single etching loop mainly consists of the three following steps: protection polymer deposition, bottom polymer breakthrough, and depth direction etching. Initially, the protection deposition step is conducted by introducing repeatable flows of C_4_F_8_ up to the values of 60 SCCM to form a thick polymer layer on both the metal NPs and the pore sidewall. Such thick polymer is wrapped on the metal NPs, which is beneficial to avoid the mask undercut and achieve a high selection ratio between the particle mask and the silicon. For the subsequent deposition step, a C_4_F_8_ flow rate of 5 SCCM is selected to form a thin protection polymer at the bottom of the pore. In the meantime, the SF_6_ flow rate of 160 SCCM is set to avoid fast C_4_F_8_ deposition to block the nanowires’ interval spacing. Such balance between the deposit and the etching procedures is adjustable according to the structure pitch size. Next, the bottom breakthrough step aims to dissolve the protective polymer and expose the silicon substrate at the pore bottom. Finally, the depth-directional etching occurs, where the posterior region of the silicon reacts with SF_6_ faster than that on the sidewall, thus realizing a highly anisotropic etching. Besides, a silicon wafer with Au film deposited on its top surface is also selected as the platform to support the silicon pieces to be etched. The platform temperature is retained constant at the value of 5 Celsius by flowing helium gas. We also used the highly thermal conductivity silicone grease to bond the etching pieces on the table silicon wafer. The goal was to accurately control the uniform piece temperature distribution and to be consistent with the set value of the platform temperature.

### 2.3. Surface Characterization

The surface texture morphology was investigated using a scanning electron microscope (SEM, Hitachi UHR FE-SEM SU8230, Hitachi, Tokyo, Japan). The reflectance and transmittance spectra were measured using a UV-Vis-NIR spectrophotometer (Shimadzu UV 3600 plus, Shimadzu, Kyoto, Japan). The BaSO_4_ compound was used as the reflectance spectrum reference from the nanowire surface, while the aluminum mirror was the reference for reflectance spectra of the polished silicon wafer. The total reflection, diffuse, and mirror reflection were measured in the integrating sphere. Furthermore, the contact angle of the nanowire texture was characterized by using the contact angle meter, which is equipped with a rotatable substrate holder and automated dispenser (CAM-01A, GLOBAL ANALYTICAL, Ankara, Turkey). The mechanical stability experiment was performed by using an automatic dripping device. Five thousand water droplets of twenty microliters were dripped continuously from a distance of 30 cm to strike the silicon surface. The bouncing effect of the water droplet on such a surface was captured and recorded by employing a high-speed video camera (5KF10, Fuhuang Agile Device, Hefei, China).

## 3. Results and Discussion

**Figure 2:**
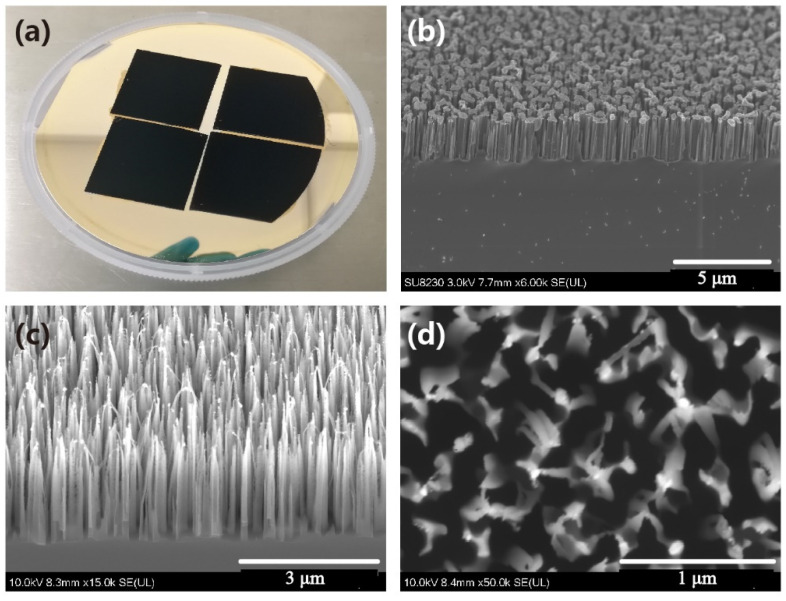
Photograph and SEM images of the silicon nanowires. (**a**) Photograph; (**b**) nanowires with Au nanoparticles on tip top; (**c**) tilted image of the nanowires formed through 120 loops of Bosch process; (**d**) top view of the nanowires formed through 120 loops of Bosch process.

Figure 2a divulges the digital photo of the samples with nanowires formed by applying 120 Bosch etching loops. The NPs were created by enforcing an annealing step on a 4 nm Au thick film and served the role of the etching mask. The sample surface exhibits a black visual due to the nanowires’ “light trapping” ability [26,27]. This ability originates from the energy decay in the surface light reflections and the optical path length extension through the internal refraction and transmission processes [26,28]. The surface reflection loss stems from both the mirror reflection effect at the smooth substrate and diffusion reflection caused by structures with small aspect ratios. As a result, the employment of surface textures with a configuration of the pore, tip, and deep holes can effectively suppress such reflection losses by permitting more incident light to enter the structure and multi-reflect along the depth direction. As far as the optical path length is concerned, it is significantly larger than the actual substrate thickness. More specifically, it is defined as the distance that an unabsorbed photon could travel within the silicon bulk before it escapes, which implies that light waves will have more opportunities to achieve a coupling decay with silicon [29]. The employment of a structure that induces multiple internal reflections, light refraction, and transmission effects can facilitate such a coupling decay process [30]. As a result, surface structures with a smaller smooth area on top and larger aspect ratios can trap an enhanced incident light, leading to constant attenuation through the consecutive interactions of light and silicon within the structure. Figure 2b illustrates the tilted SEM image of the fabricated nanowire array, showing that the Au NPs are distributed uniformly at the top of the nanowires. It is also indicated that the etching step’s depth direction has a high selection ratio between silicon and mask. In addition, the employed etching process is highly anisotropic, which is desired to avoid mask undercut and create structures with elevated aspect ratios. As depicted in Figure 2c, the fabricated nanowires with a height of ~3 μm exhibit a needle-like configuration and have average aspect ratios of about 10:1. Figure 2d divulges the top view image of the silicon nanowires, which indicates a large pore proportion that allows a relatively considerable amount of light to enter and a tiny flat surface proportion on the top to avoid the specular reflection loss of the incident light. In addition, Figure S1 illustrated the digital photographs of a 4 inch wafer of the silicon nanowires formed through 120 etching loops, indicating a good uniformity across the wafer. Table S1 illustrates the etch rate, scallop height, and profile control ability in detail. Figure S2 shows the SEM image at high magnification, which indicates that the sidewalls are smooth and the Bosch scallops have an average height of ~25 nm.

**Figure 3:**
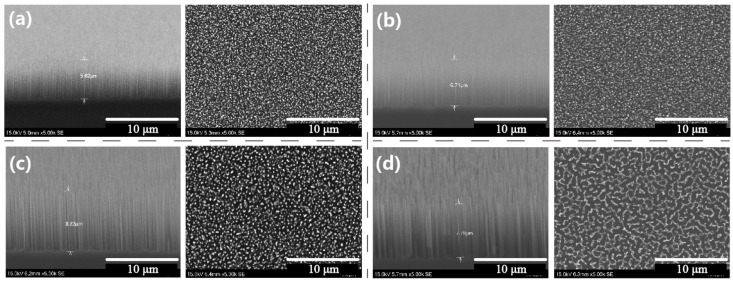
Photograph and SEM images of the silicon nanowires formed through longer etching duration and the varying Au nanoparticle sizes and etching loops. (**a**) Tilted view and top view of the nanowire formed through 160 etching loops, the particle mask is formed by 4 nm Au film; (**b**–**d**) tilted view and top view of the nanowires formed through 160 etching loops; the particle masks are formed by 6, 8, and 10 nm Au film, respectively.

To investigate the impact of the mask size and the duration of the etching process on the silicon nanowire formation, we closely examined the modifications induced by adjusting one etching loop or by employing three different particle sizes. First, Figure S3 illustrates the SEM images of the nanoparticle mask formation before the etching treatment. The Au film with deposition thickness of 4 nm was used, and it presents as continuous islands with spacings. Then, two temperatures of thermal annealing treatment were applied to compare the particle mask formation. It is indicated that the nanowire pitch size mainly depends on the spacing between the isolated particles caused by film thickness and annealing temperature. The nanowires’ diameters rely on the randomly formed particles’ sizes. Figure 3 illustrates the SEM images of the silicon nanowires formed, enforcing longer etching durations and varying the Au nanoparticle sizes. More specifically, Figure 3a reveals the tilted view and top view images of the nanowire structure formed by applying 160 etching loops and the deposition of a 4 nm thick Au film to create the particle mask. The acquired nanowires exhibited an average height of about 5.62 um, indicating that a longer etching duration produces nanowires with higher heights. Besides, Figure 3b–d illustrates the tilted and top views images of the silicon nanowires that are etched through the incorporation of particle masks formed by employing 6, 8, and 10 nm thick Au films, respectively. The obtained average nanowire heights are 6.71, 8.83, and 7.78 μm, respectively. The surface density decreases by increasing the particle size, whereas the silicon nanowire formed by 4 nm thick Au film exhibits the highest density. Since a thicker film is more likely to lead to larger NPs, the inter-particle intervals become larger as well. This effect is nicely captured in Figure 3d. Figure S4 illustrates the SEM images at high magnification of the nanowires formed through 160 etching loops; the particle masks are formed by 4, 6, 8, and 10 nm Au film, respectively. It is indicated that the silicon nanowire formed by 4 nm Au film has the highest structure density. The digital images of the nanowire-based samples are presented by employing four different particle masks formed by the deposition of 4, 6, 8, and 10 nm thick Au film. The nanowire surface induced by the presence of 4 nm thick Au film exhibits the darkest appearance, which is obvious through the digital photograph comparison illustrated in Figure S5. Thus, these silicon nanowires were selected for the following test of the optical character.

**Figure 4:**
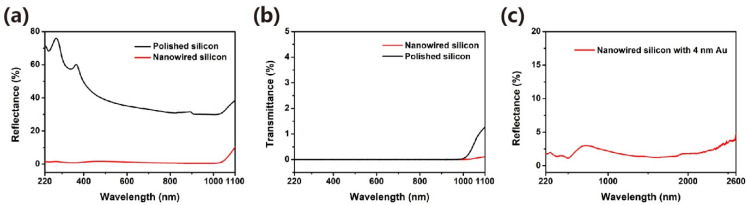
(**a**) The reflectance spectra of the polished silicon surface and the nanowire one; (**b**) the transmittance spectra of the polished silicon surface and the nanowire one; (**c**) the reflectance spectra of the nanowire silicon surface decorated with the Au nanoparticles that are formed by 4 nm Au film.

The optical measurements were carried out to investigate the nanowires’ ability to suppress the light reflection. Firstly, a polished silicon wafer was selected as a reference sample for the reflectance and transmittance spectra. Figure 4a demonstrates that the reflectance of the nanowire surface is significantly lower than that of the polished silicon wafer in the wavelength range of 220 to 1100 nm. In addition, the reflectance efficiency of the nanowire sample below the wavelength value of 1000 nm is less than 2%. Figure 4b discloses the comparative spectra, as far as the transmittance property is concerned, indicating that nanowires-based surface exhibits a lower value than the polished one at the wavelength range above 1000 nm. Such a discrepancy could be originated by the change in the material’s surface induced by the residuals of the etching reaction. Besides, we also conducted the light reflection comparison of two silicon nanowire samples formed by 4 nm film and two different etching durations. As shown in Figure S6, the silicon nanowire surface formed through 120 etch loops exhibited higher reflection values in the wavelength range of above 1200 nm than that formed through 160 etch loops. In addition, the well-known localized surface plasmon resonance (LSPR) effect [31,32,33] was applied to decrease the reflectance coupling of the near-infrared light on such nanowires-based surfaces. Au NPs were employed to generate the LSPR. The used LSPR NPs were also loaded via a DC magnetron sputtering deposition of Au, followed by a thermal annealing treatment. As divulged in Figure 4c, the nanowires decorated with Au NPs showed a lower than 3% reflectance at the wavelength range from 220 to 2600 nm.

**Figure 5:**
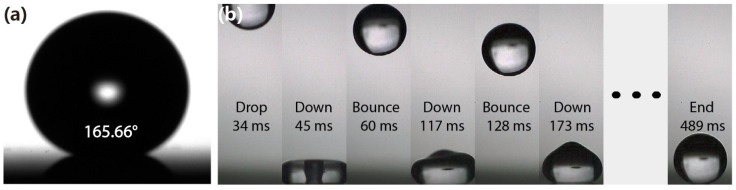
(**a**) Contact angle of the silicon nanowire surface; (**b**) screenshots of a water droplet of 1.5 μL drop onto the surface and bounce ten times, the video frame rate was 4000 per second.

It is well established that the nanowires are under intensive research for photoelectronic and energy storage-related applications due to their enhanced properties in terms of increased anti-reflectivity, high surface-to-volume ratio, and quantum confinement effects. Besides, silicon nanowires also exhibit unique structural properties that render them quietly different from the bulk silicon. More specifically, they can remarkably enhance the hydrophobicity property of the substrate surface [34,35]. The hydrophobic characteristics are of great importance since they can be utilized in the self-cleaning process, anti-bacterial surfaces, and other biomedical applications. In addition, the octadecyl trichlorosilane (OTS) is frequently applied on nanowire surface as a chemical treatment method to enhance structure-based hydrophobicity. However, such types of treatment tend to increase the contamination levels, requires high cost, and presents poor long-term reliability. It is interesting that our fabricated silicon nanowire arrays exhibited extremely superhydrophobic behavior with a contact angle over the value of ~165.6° and without implementing any hydrophobic chemical treatment, as is revealed in Figure 5a. Such a property permits the water droplet to bounce off the surface and does not allow the pendant-drop measurement to be conducted. Besides, Figure S7 shows the contact angle of the silicon nanowire surface formed by 4 nm Au film and 180 etch loops. Therefore, a hydrophobic-treated micropipette was utilized to enable the execution of the contact angle measurement by releasing a water droplet on such a surface. As illustrated in Figure 5b, the acquired screenshots from a high-speed video (the video frame rate was set to 4000 per second) demonstrate that a water droplet of 1.5 μL drops onto the nanowire surface at the height of 70 mm. Initially, it fully bounces off and consequently continues nine times to rebound along with the vertex’s height gradually decreased. The whole process has been recorded by the provided Videos S1 and S2, where the bouncing effect of a water droplet with 1.5 μL on horizontal and inclined nanowire surfaces is presented, respectively. Besides, five thousand water droplets of 20 μL drip continuously struck the silicon nanowires from 30 cm, as depicted in Figure S8, where no apparent damage of the nanowires’ surface was detected.

## 4. Conclusions

A novel DRIE method was demonstrated in this work to create silicon nanowires with controllable surface densities and high aspect ratios. Such a process involves employing an Au NPs-based deposition mask, a modified Bosch etching process, and a mask removal step. As a result, the fabricated silicon nanowire array exhibited an excellent anti-reflection below 2% within a broad light wave range from 220 to 1100 nm. Furthermore, by leveraging the formation of surface plasmons, induced by the creation of Au NPs through the dewetting process of 4 nm thick gold film, a near-unity reflectance of below 3% in the broadband of 220 to 2600 nm was obtained. Besides, our prototypes exhibited extremely super-hydrophobic properties with a contact angle over ~165.6° without applying any hydrophobic chemical treatment, resulting in bouncing off the surface properties of the water droplets. These results render the fabricated silicon nanowire a pretty promising solution for various applications, including developing novel photothermal [5,6,36,37] and photocatalysis processes [38,39], supercapacitor electrodes [40,41,42], and microfluidic devices [43,44,45].

## Figures and Tables

**Table 1:** Nanoparticles-assisted Bosch process.

Main Steps in 1 Etching Loop	SF_6_ Gas Flow (sccm)	C_4_F_8_ Gas Flow (sccm)	ICP Power (W)	HF Power (W)	O_2_ Gas Flow (sccm)	Table Temperature (°C)	Pressure (mTorr)	Helium Backing (Torr)	Step Time (ms)
Pre-deposition	10	200	1500	5	0	5	0	10	25
Deposition	5	60	1250	5	0	5	20	10	550
Deposition Sub 1	20	60	1250	5	0	5	20	10	50
Deposition Sub 2	160	60	1250	5	0	5	30	10	100
Deposition Sub 3	160	5	2000	5	0	5	30	10	50
Deposition Sub 4	160	5	2000	60	0	5	30	10	50
Breakthrough	200	5	2000	60	0	5	30	10	325
Breakthrough sub 1	200	5	2000	60	0	5	30	10	100
Breakthrough Sub 2	200	5	2500	60	0	5	80	10	50
Breakthrough Sub 3	200	5	2500	0	0	5	80	10	50
Etch	500	5	2500	0	0	5	80	10	600
Etch Sub 1	1	120	2500	0	0	5	80	10	150
Etch Sub 2	5	120	1250	0	0	5	20	10	100

## Data Availability

Data are contained within the article.

## Supplementary Materials

The following are available online at https://www.mdpi.com/article/10.3390/mi12091009/s1, Figure S1: Digital photographs of the 4-inch wafer of the silicon nanowires formed through 120 etching loops; Figure S2: The SEM image at high magnification, indicating the smooth sidewalls and the Bosch scallops with the height of ~25 nm; Figure S3: The SEM images of the nanoparticle masks before etching treatment; Figure S4: SEM images at high magnification of the nanowires formed through 160 etching loops, the particle masks are formed by 4, 6, 8, and 10 nm Au film, respectively; Figure S5: Digital photographs of the nanowires formed through 160 etching loops, the particle masks are formed by 4, 6, 8, and 10 nm Au film, respectively; Figure S6: The reflectance spectra of the polished silicon surface and the nanowire ones formed by 4 nm film. The nanowires are formed through 120 and 160 etch loops, respectively, while the planar silicon is used as the reflectance. reference; Figure S7: Contact angle of the silicon nanowire surface formed by 4 nm Au film and 180 etching loops; Figure S8: SEM image of the tilted view of the silicon nanowire array after striking by five thousand water droplets continuously; Table S1: Nanoparticles-assisted Bosch process; Video S1: the whole process of the water droplet with 1.5 μL bouncing on the horizontal nanowire surface; Video S2: the whole process of the water droplet with 1.5 μL bouncing on the inclined nanowire surface.
