# Supplementary material for: Fabrication of Needle-Like Silicon Nanowires by Using a Nanoparticles-Assisted Bosch Process for Both High Hydrophobicity and Anti-Reflection
Source: Micromachines (Basel). 2021 Aug 25;12(9):1009. doi: 10.3390/mi12091009 (PMC8471489; doi:10.3390/mi12091009)
Supplement: Supplementary file 1 [file micromachines-12-01009-s001.zip › micromachines-1343201-supplementary.pdf]

Supporting Information

Fabrication of needle-like silicon nanowires by using a nano-particles-assisted Bosch process for both high hydrophobicity and anti-reflection

Zengxing Zhang [1], Guohua Liu [2] and Kaiying Wang [1,*]

1 Department of microsystems, University of South-Eastern Norway, Horten, 3184, Norway; zengxingzhang88@163.com
2 Beijing Key Laboratory of Multiphase Flow and Heat Transfer for Low Grade Energy Utilization, North China Electric Power University, Beijing, 102206, China; liuguohua126@126.com
* Correspondence: kaiying.wang@usn.no

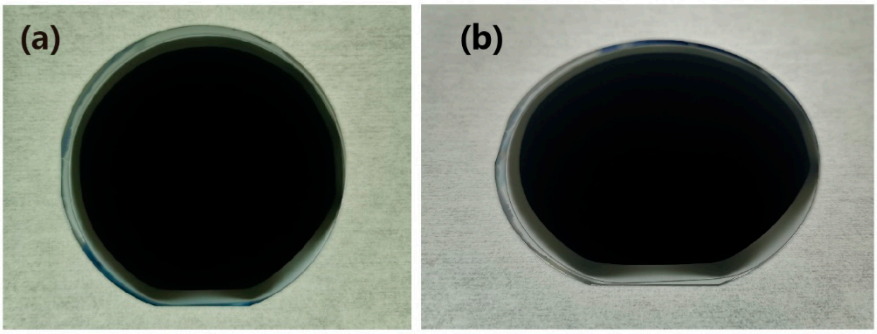

Figure S1. Digital photographs of the 4-inch wafer of the silicon nanowires formed through 120 etching loops. (**a**) Top view, (**b**) Tilted view.

Table S1. Nanoparticles-assisted Bosch process.

Items	Sample-120 etching loops
Etch depth	3~3.5 um
Etch rate / um/min	~0.75 um/min
Scallops / nm	~25 nm
Profile control	90±1º
Wafer uniformity	Good

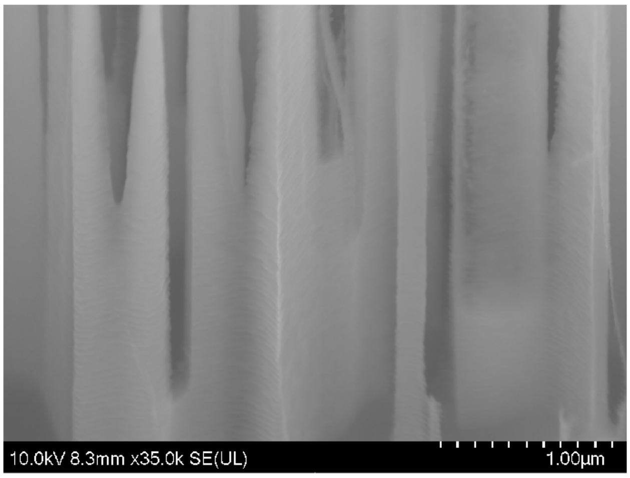

Figure S2. The SEM image at high magnification, indicating the smooth sidewalls and the Bosch scallops with the height of ~25 nm.

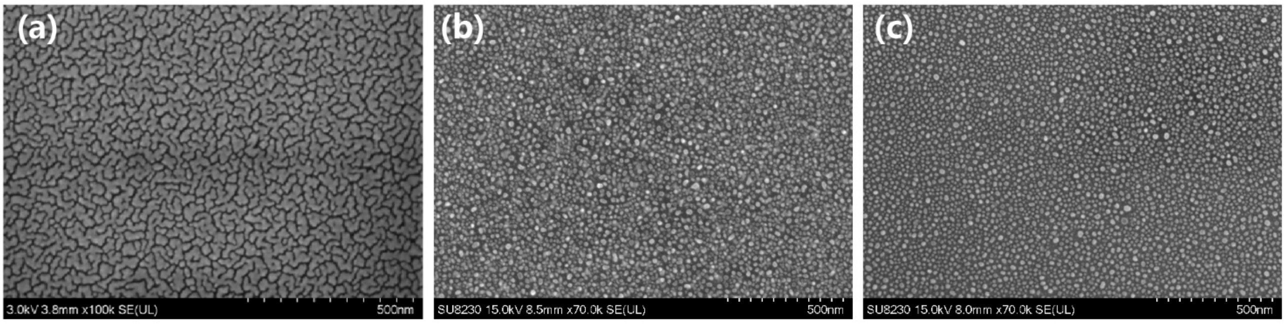

Figure S3. The SEM images of the nanoparticle masks before etching treatment. (**a**) The 4 nm Au film without thermal annealing treatment. (**b**) The nanoparticles formed by 4 nm Au film and thermal annealing at 500 ℃ for one hour. (**c**) The nanoparticles formed by 4 nm Au film and thermal annealing at 600 ℃ for one hour.

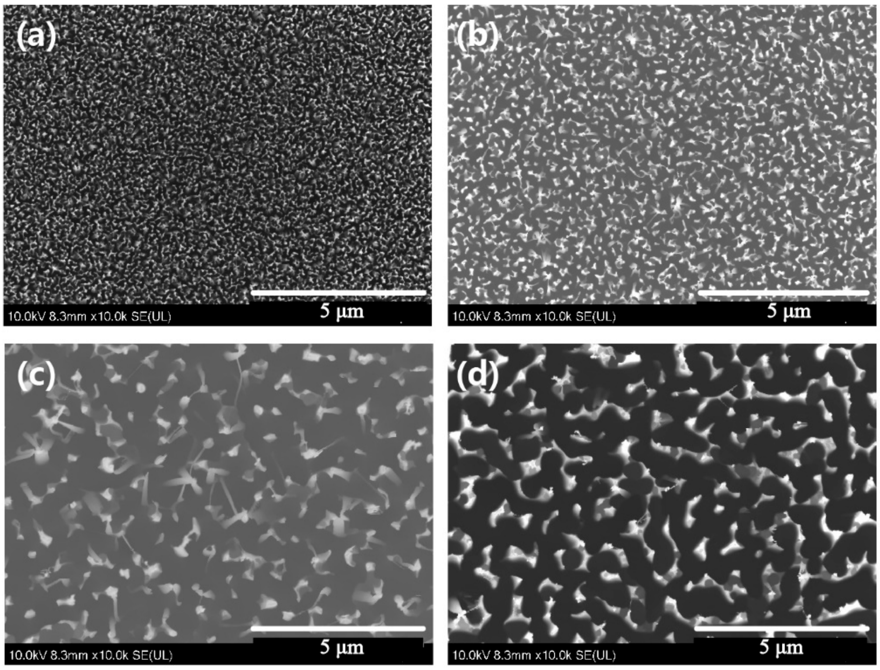

Figure S4. (**a**–**d**) SEM images at high magnification of the nanowires formed through 160 etching loops, the particle masks are formed by 4, 6, 8, and 10 nm Au film, respectively.

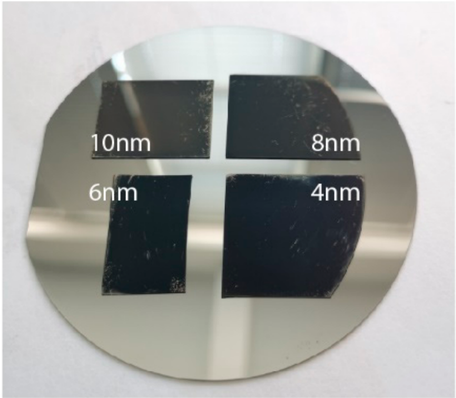

Figure S5. Digital photographs of the nanowires formed through 160 etching loops, the particle masks are formed by 4, 6, 8, and 10 nm Au film, respectively.

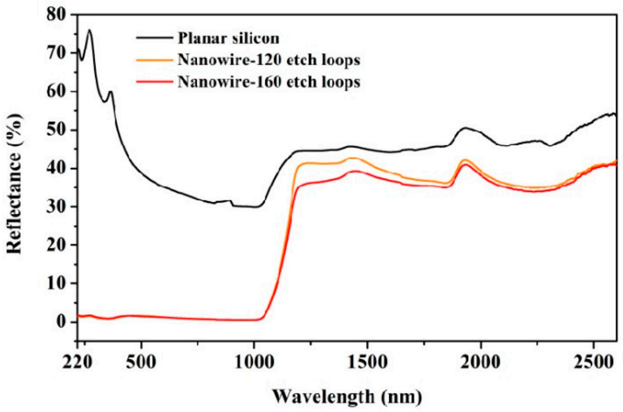

Figure S6. The reflectance spectra of the polished silicon surface and the nanowire ones formed by 4 nm film. The nanowires are formed through 120 and 160 etch loops, respectively, while the planar silicon is used as the reflectance. reference.

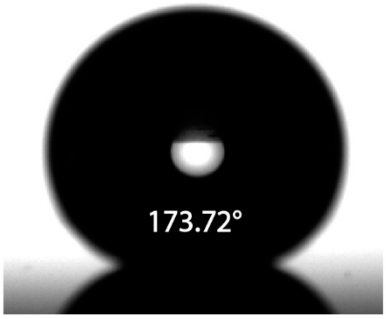

Figure S7. Contact angle of the silicon nanowire surface formed by 4 nm Au film and 180 etching loops.

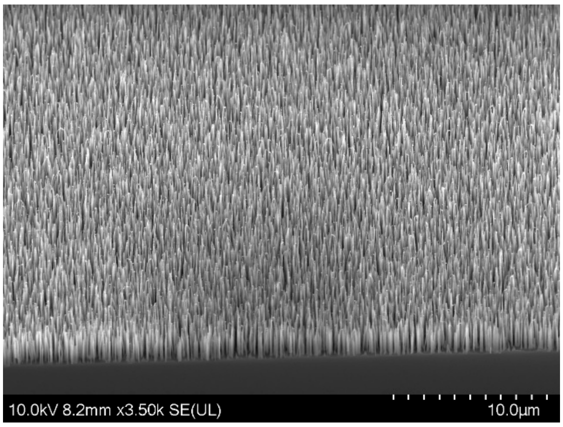

Figure S8. SEM image of the tilted view of the silicon nanowire array after striking by five thousand water droplets continuously.

Video S1. The whole process of the water droplet with 1.5 μl bouncing on the horizontal nanowire surface.

Video S2. The whole process of the water droplet with 1.5 μl bouncing on the inclined nanowire surface.
